# Local Coordination Environment of 3d and 4d Transition Metal Ions in LiCl-KCl Eutectic Mixture

**DOI:** 10.3390/ma15041478

**Published:** 2022-02-16

**Authors:** Jon Fuller, William Phillips, Qi An, Ruchi Gakhar

**Affiliations:** 1 Idaho National Laboratory, Pyrochemistry and Molten Salt Systems Department, Idaho Falls, ID 83415, USA; jonfuller@nevada.unr.edu (J.F.); william.phillips@inl.gov (W.P.); 2 Department of Chemical and Materials Engineering, University of Nevada, Reno, NV 89577, USA

**Keywords:** HSE, DFT, molten salts, LiCl-KCl, optical absorption spectroscopy

## Abstract

In this study, the structure and coordination environment of two 3d transition elements (Ni and Cr) is investigated in a molten chloride salt system. Electronic absorption spectroscopy was employed to elucidate their coordination environment in 3LiCl-2KCl eutectic salt, as a function of temperature. Density functional theory (DFT) modeling was used to determine the coordination environment of the transition metal species in the eutectic composition as well as the optical spectra computationally. The Ni^2+^and Cr^3+^ exist in a tetrahedral and octahedral coordination environment, respectively, in eutectic salt. The spectra thus obtained were compared with the experimental data; a reasonable qualitative agreement was obtained between experimental and computational Ni^2+^ and Cr^3+^spectra, and the coordination of both elements in the eutectic composition were in excellent agreement with the experimentally determined results. Computational results were also obtained for two 4d elements, Mo^3+^ and Nb^3+^, with both quantum molecular dynamics (QMD) and hybrid functional optical spectra indicating octahedral coordination.

## 1. Introduction

The coordination chemistry of various metal species in different molten salt compositions has become a very important focus for research development in molten salts, in order to discover the nature of their chlorocomplex formation mechanisms and their relationships with chemical processes, which are relevant to the functionality of molten salts applications, such as corrosion, electrolysis effects and other chemical processes [1,2]. Particularly, molten salt-based pyroprocessing technology is the key process for treating the used nuclear fuels to close the nuclear fuel cycle [3]. The thermodynamic and electrochemical properties of actinides and other fission products in eutectic molten LiCl-KCl salt are pivotal to optimize, monitor and control the pyroprocessing process [4]. The coordination chemistry of these metal complexes in the melt are intrinsically linked to several important properties for the molten salt, including reactivity, diffusion rates and thermophysical behavior with other species, particularly actinides, encountered during the pyroprocessing operation [5].

Metal ions in chloride salts have more complex bonding configurations than fluorides in general because of the additional oxidation states (+7, +5, +3, +1, and −1) available for chlorine [6], leading to intriguing possible structural properties and coordination environments. The metal complex in chloride salts may have a significant impact on the salts properties that are important for their engineering applications. The most pertinent information concerning the coordination behavior of actinides in molten salts can be obtained using electronic absorption spectroscopy [7,8]. For investigation of the structural symmetry and chemical constitution of molten salts for application to electrorefining and molten salt reactor (MSR) applications, a versatile high-temperature apparatus for in situ spectroscopic studies of molten salts has been designed at the Idaho National Laboratory (INL), with a capability to combine several spectroscopy and electrochemical techniques simultaneously (discussed later in Section 2.1) [9]. 

The optical absorption spectra of transition metal ions have been analyzed experimentally in several molten halide salts [10,11,12,13,14,15,16]; however, the absorption spectra are not usually accurately obtained and compared using a computational framework [17,18,19]. Computational analysis to predict various properties of the salt systems, especially coordination and structural chemistry, is an area with widening interest for metal inclusions in various molten salt media. Molten salts pose a unique challenge computationally for density functional theory (DFT) methods, as the complexity of the system is relatively high, even for binary salt compositions, such as LiCl-KCl. This complexity hampers more resource-intensive methods and requires novel adaptation of existing techniques to provide meaningful results. It is relatively well known that excitonic effects play a significant role in optical spectra calculations [20,21,22]. However, performing Bethe–Salpeter equation calculations (which explicitly treat exitonic effects through a quasiparticle approximation using either the Green–Coulomb approximation (GW) method or the density functional perturbation theory (DFPT) method) [23] are extremely computationally intensive, especially for complex systems, such as a molten salt. 

Here, we qualitatively predict the coordination behavior of the selected transition metal ions in the eutectic composition using computational methods combining quantum molecular dynamics (QMD) and the Heyd–Scuseria–Ernzerhof exchange-correlation functional (HSE-06) optical calculations, in order to correlate the coordination of transition metal ion and absorption spectra with the experimentally obtained data. To the best of the authors’ knowledge, calculation of optical absorption spectra from transition metals in molten chloride melts using hybrid-functionals has not been investigated and reported. A detailed comparison with the experimentally obtained spectra for 3d transition metal ions allowed the authors to determine the applicability of hybrid functional-based calculations, on which the optical spectra of two 4d transition metal ions were computed. The challenges, strengths and shortcomings of two computational approaches, Perdew–Burke–Ernzerhoff (PBE) and the HSE-06 hybrid functional for the prediction of optical spectra and the coordination environment of metal species have been discussed. This experimentally validated computational approach can be applied to various salt systems to investigate the structure and coordination environment in an extended wavelength range and temperature regime. 

## 2. Materials and Methods

### 2.1. Apparatus

All operations pertaining to the handling of the molten salt compositions and optical spectroscopy measurements were performed inside a glovebox containing an Ar atmosphere (Vacuum Technologies Inc., Gloucester, MA, USA) maintained at less than 0.1 ppm moisture and oxygen concentrations. The optical absorption spectra were recorded using a Cary 5000 spectrophotometer, equipped with a deuterium and tungsten lamp used to cover wavelengths across the ultraviolet to near infrared region from 200 to 2300 nm. The apparatus used for collecting the experimental data has been described in a previous publication [9]. The wavelength scale of the spectrophotometer in the range of 880 to 280 nm was verified and calibrated using a National Institute of Standards and Technology certified reference material consisting of an aqueous solution prepared from didymium perchlorate, which is permanently inside a high quality far ultraviolet (UV) quartz cell, certificate number 72,641.

### 2.2. Experimental Methods

**Figure 1:**
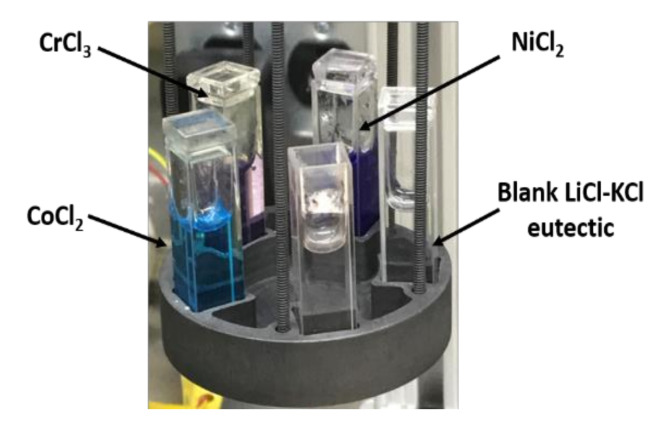
Molten LiCl-KCl eutectic salt solution immediately after withdrawal from the heating furnace at 500 °C containing NiCl_2_ (purple color) and CrCl_3_ (pinkish purple) in quartz cuvette, along with the blank LiCl-KCl eutectic (clear). Solution containing CoCl_2_ is shown for color comparison (blue).

Anhydrous LiCl-KCl eutectic, NiCl_2_ and CrCl_3_ of 99.99% purity (trace metals basis) were procured from Sigma–Aldrich (Burlington, MA, USA) and were used as received (no further salt purification was performed). The quartz cuvettes were rinsed with acetone and isopropanol, then dried under an evacuated Ar atmosphere at 175 °C overnight prior to use. Custom designed quartz lids were used to minimize salt vaporization at elevated temperatures and were subjected to the same cleaning procedure as the cuvettes. Samples were prepared by introducing the desired masses of salts in the cleaned and dried cuvettes at room temperature, prior to loading into the furnace. Sample weights were recorded to 0.1 mg precision using a Mettler Toledo balance. Due to the differences in the molar absorptivity of NiCl and CrCl_3_, the concentrations were adjusted to provide the highest signal to noise ratio possible without overwhelming the detection limits of the spectrophotometer. The total salt mass for each sample was approximately 5 g. The NiCl_2_ and CrCl_3_ were loaded in 0.1 wt% concentrations in the LiCl-KCl eutectic, respectively. A sample of blank LiCl-KCl eutectic salt was used to record the baseline spectra prior to recording spectra of each species at each temperature point. The design of the furnace allowed the parallel run for all the samples at each temperature setpoint. The transition metal chlorides were allowed to dissolve in LiCl-KCl melt at 600 °C to ensure complete/maximum dissolution; spectra were then recorded on the cool down from 600 °C to 400 °C at intervals of 50 °C. At each temperature set point, the furnace was allowed to equilibrate for 30 min prior to recording the baseline and subsequent sample spectra. These samples are shown in their molten form in Figure 1.

### 2.3. Computational Methods

All calculations in this work were performed using the Vienna Ab-initio Simulation Package (VASP) code [24,25,26,27]. A supercell was devised to represent the LiCl-KCl eutectic composition (59.2% mol LiCl) comprised of 19 Li atoms, 32 Cl atoms, and 13 K atoms, which is shown in Figure S1 of the Supplemental Materials (SM). The initial structure was constructed based on experimental density [14] and the atoms are randomly distributed in the supercell. Then, quantum molecular dynamics (QMD) was performed using the PBE functional [28,29] to optimize the structure through a rapid quenching simulation beginning with heating to T = 2000 K (a temperature well above the melting point) for 5 ps to ensure the constituent atoms in the simulation reached sufficient equilibrium for liquid behavior prior to further refinement. Each system was then “quenched” in the simulation from 2000 K to the target temperature rapidly over a period of an additional 5 ps. Finally, each system was equilibrated for a longer period of time (with a minimum of 10 ps) at the fixed target temperature. Equilibration was performed continuously with adjusting volume in all cases until the pressure of the system converged to near zero and the energy of the system was deemed to be converged. Figure S1 shows the energy convergence for the Ni^2+^ in the simulated LiCl-KCl eutectic as an example. Figure S2 shows the optimized LiCl-KCl eutectic system after equilibration, as described above.

**Figure 2:**
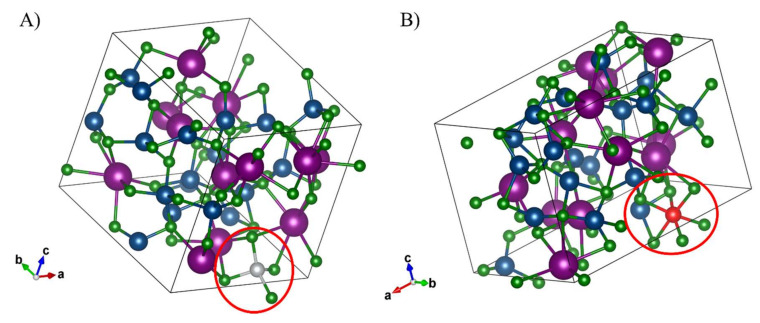
The transition metal chlorocomplex is circled in red, with the 3d cation shown in grey (Ni) or red (Cr) and Li/Cl/K shown in blue, green and purple, respectively. Configuration of (**A**) coordination of NiCl_4_^2−^ chlorocomplex and (**B**) CrCl_6_^3−^ chlorocomplex, in LiCl-KCl eutectic composition at 400 °C. The chlorocomplex is circled in red in each case for clarity.

In order to simulate the inclusion of the three transition metals targeted in this study, a corresponding quantity of K and Li atoms were removed and replaced with the transition metal cation prior to the high-temperature simulation in order to preserve the nearest-possible composition to the eutectic. These systems were quenched to temperatures of 400 °C, 500 °C and 600 °C then equilibrated at the target temperature in order to allow direct comparison with the experimentally obtained results for each transition metal-molten salt system. Additionally, in order for the proper bonding behavior to be exhibited in the QMD, each calculation containing a metal cation was treated with spin polarization specified to ensure accurate results. Ni and Cr were treated with the per-atom magnetic moment of 0.6 and 0.7 μB, respectively. The energy cutoff for the wavefunction expansion was specified to be 400 eV. The self-consistent field (SCF) convergence criteria were designated as 1 × 10^−5^ eV with a maximum number of 200 cycles. Normal precision was used for computational simplicity due to the complexity of the simulated system. Fermi smearing was applied to all QMD calculations with a smearing width of 0.172 eV. All visualization of QMD results, including Figure 2 and Figure S2 were performed using the VESTA software (Koichi Momma, Ibaraki, Japan) [30].

Optical spectrum calculations were performed using the VASP calculation method explained by Gadjos et al. [31], in which a Kubo–Greenwood style linear response was calculated for both the real and imaginary portions of the dielectric function, with the imaginary portion being used to calculate optical properties, including absorption. The imaginary portion is calculated according to the following equation:

(1) $$\varepsilon \frac{im}{\alpha \beta }\left(\omega \right)=\frac{4\pi^{2}e^{2}}{\Omega }\underset{q\to 0}{\lim}\frac{1}{q^{2}}\sum c,v,k\;2W_{k}\delta \left(\epsilon_{ck}-\epsilon_{vk}-\omega \right)\times \langle U_{ck+e_{\alpha }q}|U_{vk}\rangle \langle U_{ck+e_{\beta }q}|U_{vk}\rangle$$

The details of the derivation of the absorption spectrum from the real and imaginary dielectric tensor components are explained in detail in previous work [31]. In Equation (1), c represents the conduction band states specified in initial input for the calculation, while v indicates the valence band states for the system obtained from the band structure calculation and k represents the *k*-point for this specific calculation. This means that, as the imaginary dielectric function is evaluated at each *k*-point for conduction and valence bands c and v, increasing any of these three values by evaluating a denser *k*-point mesh or specifying more accurate valence and conduction band settings (approaching the total number of bands, NBANDS) drastically increases the computational requirements of the calculation. In this calculation method the NBANDS value was increased to three times the total valence electrons in the simulated system in order to allow for a better calculation of the dielectric tensor, while the values of c and v were kept to the minimal values in order to lower the computational requirements.

Optical calculations were carried out according to a two-part schema, in which the ground state band structure and wave functions for the target system were calculated in a single calculation, followed by an optical calculation to derive the frequency-dependent dielectric tensor, as described previously. The VASPKIT (Wang et. al) post-processing software [32] was used to derive the absorption spectra for each species.

For the optical calculations, both the PBE functional and the HSE-06 functional were evaluated. The PBE functional was found to be unsatisfactory for use due to inaccurate band structure calculations stemming from underestimation of the band gap for complex systems. The hybrid HSE-06 [33] functional, using a 25/75 mix of Hartree–Fock and DFT calculations, was found to result in a significantly more accurate absorption spectrum for each species relative to the experimentally observed spectrum than the PBE functional, due to its more accurate band structure calculation for each system leading to a more-accurate dielectric tensor obtained from the resulting wave functions. Similarly, the k-points mesh was chosen to be the relatively simple gamma-centered Monkhorst-Pack 2 × 2 × 2 configuration in order to minimize computational requirements due to the complexity of the unit cell, as finer *k*-point meshes were found to be prohibitively computationally expensive. Gaussian smearing was used in all calculations, and the complex shift η was approximated to be 0.100 eV.

Due to the inherent fluctuations of the molecular dynamics approach and to improve the quality of the resultant band structure for dielectric tensor computation, a trajectory average was obtained by averaging the individual positions of each atom from the QMD across a designated “timespan” of timesteps in order to obtain an averaged overall structure for the eutectic salt composition in each case. Averages obtained from individual positions across timestep spans of >0.6 ps were found to exacerbate bond angle issues with the transition metal chlorocomplexes, while <0.3 ps averages were found to vary significantly from each other over the course of the MD. As such, all optical results were obtained from averages of the individual positions from 0.6 ps (600 MD steps in VASP using the default timestep of 1 fs) of the QMD.

## 3. Results and Discussions

The LiCl-KCl solution containing NiCl_2_ and CrCl_3_ in a molten state are shown in Figure 1. The NiCl_2a_ solution is characterized as a vibrant purple color in the chloride melts, while CrCl_3_ obtains a pinkish translucent color in the melt. The blank LiCl-KCl eutectic is transparent in molten state. The color of each melt remained unchanged through the temperature range investigated.

The d-d transitions, and hence the geometrical arrangement of the central 3d metal ion, are easily influenced by the fields of the nearby ions and molecular anions, as the 3d shell electrons are only lightly protected by the outer electrons. Due to the partially filled d shells, the spectrum of transition-metal ions is characteristic of broad, weak bands in the near-IR and visible spectral regions and high intensity UV bands. The bands in the near-IR and visible range are generated by d-d transitions, or in other words, transitions within specific energy levels of d^n^ electronic configurations. The strong bands in the ultraviolet are known as charge-transfer transitions and occur due to transfer of p electrons from a ligand anion (a halide ion in this case) into an enlarged orbital that is confined by nearby cations.

**Figure 3:**
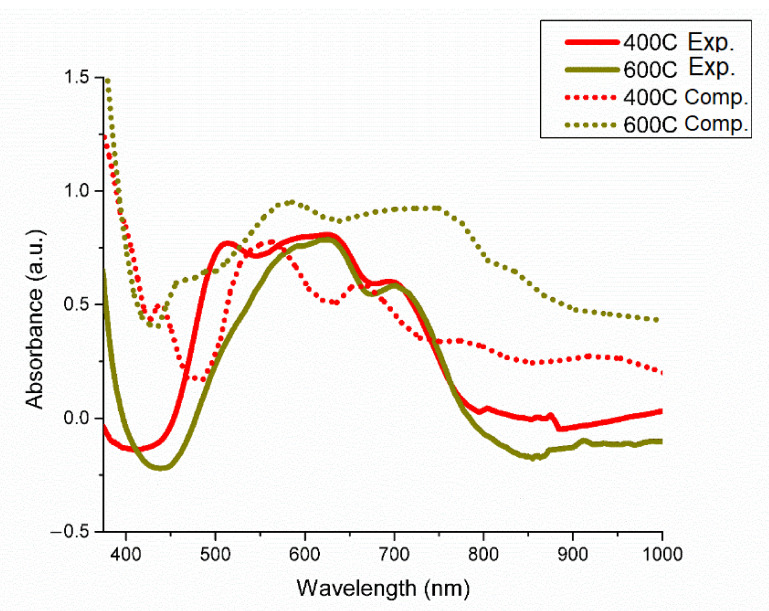
Experimental and computationally obtained optical absorption spectra for Ni^2+^ in the LiCl-KCl eutectic system as a function of temperature. Computational spectra at 400 °C and 600 °C demonstrate moderate correlation with experimental results, with expected blue shift in peak location.

**Figure 4:**
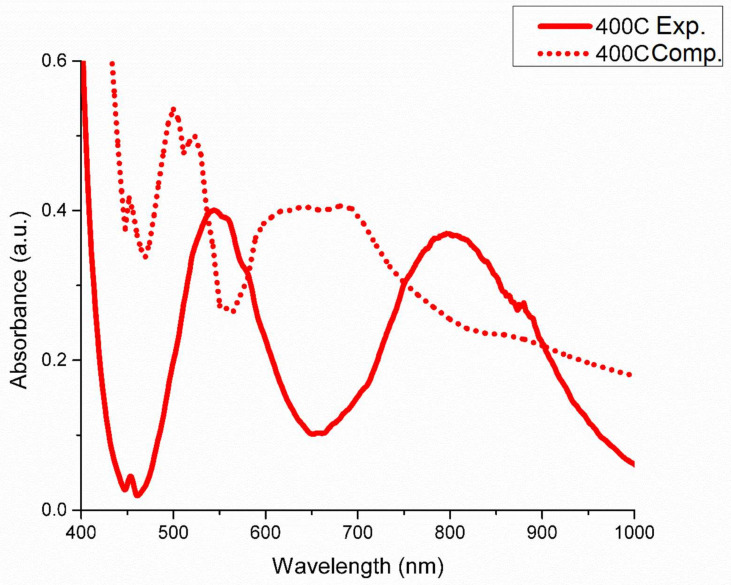
Experimental and computational optical absorption spectra of Cr^3+^ in LiCl-KCl eutectic melt at 400 °C, with the solid line representing the experimentally obtained spectrum and the dotted line representing the computational absorbed spectrum using the HSE-06 functional. Significant blue shift is evident for both peaks in the computational spectrum.

**Figure 5:**
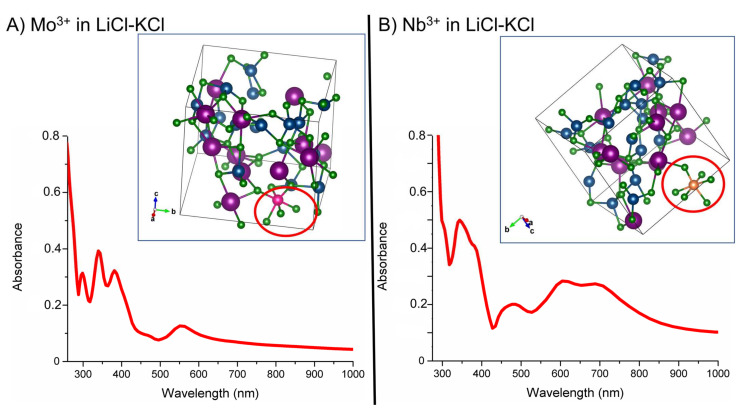
The transition metal chlorocomplex is circled in red, with the 4d cation shown as a pink (Mo) or orange (Nb) sphere, and Li/Cl/K is shown as blue, green and purple spheres, respectively. (**A**) MoCl_6_^3−^ chlorocomplex in LiCl-KCl eutectic at 400 °C. (**B**) NbCl_6_^3−^ chlorocomplex in LiCl-KCl eutectic at 400 °C. (**A**) Computationally obtained optical absorption spectra for Mo^3+^ in LiCl-KCl eutectic composition at 400 °C. (**B**) Computationally obtained optical absorption spectra for Nb^3+^ in LiCl-KCl eutectic composition at 400 °C.

The coordination of Ni^2+^ and Cr^3+^ chlorocomplexes in the LiCl-KCl eutectic solution are shown in Figure 2A,B, respectively. Ni^2+^ is found to adopt a tetrahedral coordination, while Cr^3+^ is found to adopt an octahedral coordination. The optical absorption spectra of the selected species in the LiCl-KCl eutectic media are shown in Figure 3, Figure 4 and Figure 5. For Ni^2+^ and Cr^3+^, the important charge transfer band found in the lower wavelength range was observed to shift to lower energies with increased temperature. This charge transfer band shift is similar to what has been observed for Ni^2+^ and Co^2+^ in ZnCl_2_ [12,34] and is attributed to the increased thermal energy of the system leading to a corresponding lowered energy requirement for the resulting charge transfer between Cl^−^ and the relevant cation [35].

The calculated optical absorption spectra obtained using the HSE-06 hybrid functional is likewise presented in Figure 3, Figure 4 and Figure 5. Exitonic effects, not explicitly treated in hybrid functional calculations, and their impact on the calculated optical spectra are visible in all cases, leading to blue shift of signature features for each element with varying degrees of severity along with distortion of the peak profiles. The configuration of each species obtained from QMD is shown in Figure 2, with each agreeing with the experimental results obtained here and in previous works [14]. For Ni^2+^ in the LiCl-KCl eutectic system, temperature effects were also investigated computationally to demonstrate the relation between the strengthened tetrahedral coordination at higher temperatures theorized to explain the difference in experimental spectra across various temperatures.

The optical absorption spectrum of NiCl_2_ dissolved in LiCl-KCl has been experimentally studied as a function of temperature by Boston and Smith [29], Jorgensen [16] and Gruen et al. [14] The changes in the features of the spectrum with increasing temperature have been previously assigned to the changes in the population ratio of the tetrahedral and octahedral nickel chlorocomplexes in the system. An alternative explanation for this behavior was proposed by Sundheim and Harrington [34], and was based on theoretical reasons that the spectrum primarily corresponds to the tetracoordinated nickel ions with bond angle distortion from optimal tetrahedral symmetry that increases as temperature decreases towards the melting point of the system. Based on a recent study by one of the coauthors on Ni^2+^ in ZnCl_2_ media, Ni^2+^ seems to adopt ‘distorted’ tetrahedral geometry, which is validated by both optical spectroscopy and AIMD simulations [34]. In this case as well, distorted T_d_ geometry is observed for Ni^2+^ at low temperatures. However, with increase in temperature above 450 °C, the complex transitions into a pure T_d_ environment. Such change in symmetry can be viewed as the stark decrease in the intensity of the feature around 500 nm with temperature. 

The differences in the distorted tetrahedral and ordinary tetrahedral coordination of Ni^2+^ in the form of the NiCl_4_^2−^ chlorocomplex (shown in Figure 2) are visible in our computationally obtained spectra, shown in Figure 3 and Figure S3 of the SI. Our calculations indicate the 400 °C HSE-06 spectra (dotted red line) to contain the distorted three-peak key feature shown in Figure 3, which is notably diminished in the 600 °C feature shown (dotted gold line). As with the Cr^3+^ system, a degree of blue shift tied to the exitonic effects in the system is present in the spectrum, although to a more muted degree. 

The orientation of the NiCl_4_^2−^ bond angles likewise change between the two target temperatures in the QMD, with the average Ni-Cl bond angle after equilibration at 400 °C found to be 112.4° and at 600 °C the average bond angle was found to be 110.1°, significantly closer to the ideal 109.5° for a tetrahedral complex. These results indicate a transition towards a pure tetrahedral configuration at increased temperatures, although some distortion in bond angles remains due to the forces inherent in the QMD. This supports the temperature-dependent distorted tetrahedral symmetry concept, previously proposed by Sundheim and Harrington [34].

The optical absorption spectrum of trivalent Cr has been previously studied by Harrington and Sundheim [36], and also by Gruen and McBeth [14] in the LiCl-KCl eutectic. The spectrum of trivalent Cr in LiCl·KCl was found by both groups to correspond to octahedral CrCl_6_^3−^ ions. The spectrum contains two bands in the visible region, assigned as follows: 800 nm to ^4^A_2g_(F)→^4^T_2g_(F), and 540 nm to ^4^A_2g_(F)→^4^T_1g_(F). There are no prominent features in the near-IR range. The spectral features and thus the geometry for the Cr^3+^ chlorocomplex remains consistent over the temperature range investigated. This is consistent with QMD results, shown in Figure 2A, where a strong octahedral CrCl_6_^3−^ coordination is evident at all investigated temperatures. The calculated spectrum at 400 °C is shown in Figure 4 with a dotted line and exhibits some peculiarities compared to the experimentally obtained results, likely due to bond–angle distortion from the MD and the untreated exitonic effects inherent to our calculation method. Importantly, the characteristic dual transitions corresponding to the 540 nm and 800 nm are present, although 540 nm peak is blue-shifted roughly 40 nm and the 800 nm peak is blue-shifted significantly to ~700 nm in the computational spectrum. The spectra for Cr^3+^ in LiCl-KCl eutectic were found to not be significantly temperature dependent, unlike Ni^2+^, in either computational or experimental analysis.

To further test our computational approach, we also analyzed the 4d transition metals, Mo and Nb, in the LiCl-KCl eutectic composition previously described. In Figure 5, the computed coordination of Mo^3+^ and Nb^3+^ at 400 °C is shown along with the computationally obtained optical absorbance using the HSE-06 hybrid functional. We find that both Mo and Nb obtain an octahedral structure in the eutectic melt (MoCl_6_^3−^ and NbCl_6_^3−^) at 400 °C. This observation is in good agreement with prior experimental research on the two species in the LiCl-KCl eutectic melt [37,38].

Mo can potentially adopt several coordination states due to disproportion in the LiCl-KCl eutectic, depending on a variety of conditions found under prior electrochemistry experiments, including the electrochemically important MoCl_6_^3−^ and byproduct MoCl_5_ and MoCl_4_^2−^ chlorocomplexes, both of which are believed to be highly volatile [37]. Therefore, our efforts were focused on the Mo^3+^ coordination to obtain the coordination in LiCl-KCl. The coordination of Mo^3+^ in the form of MoCl_6_^3−^ is shown in Figure 5A; the Mo cation forms an octahedral chlorocomplex with somewhat distorted bond angles from the preferred 90°, likely due to the inherent forces in the MD technique applied. The octahedral coordination is visible in the optical absorption spectra shown in Figure 5A, where a double peak feature is found reminiscent of the octahedral spectra obtained for Cr^3+^ in Figure 4. The experimental results reported by Gabriel et al. lacked a conclusive coordination state for Mo in LiCl-KCl primarily due to its high volatility. Our computational results signify the formation of a distorted octahedral complex MoCl_6_^3−^ for Mo^3+^ at 400 °C.

Likewise, Nb has also been found to adopt multiple coordination states in LiCl-KCl [38], and we have chosen to focus on the predominant Nb^3+^ coordination for our computational results. The octahedral NbCl_6_^3−^ chlorocomplex forms in the simulated eutectic, as shown in Figure 5B; the average bond angle for the chlorocomplex was found to be 92.1°, which is much closer to the ideal 90° than our results for the similarly behaving MoCl_6_^3−^. This stronger octahedral geometry for the chlorocomplex is evident in the absorption spectra shown in Figure 5B, where the two-peak feature is more readily recognizable as an octahedral spectra. We find reasonable qualitative agreement with the LiCl-KCl eutectic results reported by Brevnova et al., who similarly reported an octahedral spectra for NbCl_6_^3−^ with absorption peaks at 480 and 650–700 nm that correspond to the ^3^T_1g_→^3^T_1g_(P) and ^3^T_1g_→^3^T_2g_ transitions. We similarly find a broad feature in the 600–700 nm range paired with a narrower feature at around 475 nm, shown in Figure 5B above.

## 4. Conclusions

We report the experimental and computational optical absorption spectra (using the HSE-06 hybrid functional) for four transition metal chlorocomplexes important to pyroprocessing and corrosion processes in the LiCl-KCl binary eutectic salt composition, including those formed by Ni^2+^, Cr^3+^, Mo^3+^ and Nb^3+^ as well as their predicted coordinations using QMD. We find from the QMD that Ni^2+^ forms the tetrahedral NiCl_4_^2−^ chlorocomplex, while Cr^3+^, Mo^3+^ and Nb^3+^ form the octahedral CrCl_6_^3−^, MoCl_6_^3−^ and NbCl_6_^3−^ chlorocomplexes, respectively. The results obtained agree with previous experimental conclusions and are supported by the optical spectra obtained both experimentally and computationally in this work.

Experimental spectra for Ni^2+^ indicates a shift from distorted tetrahedral to distinct tetrahedral as the temperature increases, with distorted tetrahedral three-peak spectra found for temperatures below 450 °C and two-peak spectra found at higher temperatures; our QMD results support this, with the tetrahedral NiCl_4_^2−^ chlorocomplex forming at all temperatures and with a decrease of average bond angle towards the 109.5° tetrahedral ideal found in optimized position-averaged systems at higher temperatures. We likewise found more distinct two-peak spectra in computational optical absorption spectra at elevated temperatures. Computational optical absorption spectra for Cr^3+^, Mo^3+^ and Nb^3+^ all featured separated two-peak spectra indicative of octahedral coordinations, with Cr^3+^ showing blue shift compared to experimental results for both characteristic features. All computational optical spectra were found to have deviation from the experimental spectra, likely due to the untreated excitonic effects that require a separate quasiparticle calculation evaluated, using the Beth–Salpeter equation (BSE) method. The full BSE treatment is, however, difficult to perform for a system of this complexity due to the intense computational resource requirements. A natural development of these computational optical spectroscopy methods is finding a way to minimize the resources of such a calculation to evaluate the optical spectra of chlorocomplexes more accurately in molten salts systems.

## Data Availability

The data presented in this study are available in the supplementary materials in a .xlsx file.

## Supplementary Materials

The following supporting information can be downloaded at: https://www.mdpi.com/article/10.3390/ma15041478/s1, The SM file contains Figure S1—Energy convergence of Ni^2+^ in LiCl-KCl eutectic at 400 °C, Figure S2—LiCl-KCl eutectic composition from molecular dynamics simulations at 400 °C and Figure S3—Ni^2+^ in LiCl-KCl at 400 °C, obtained experimentally and from the HSE optical calculation. The .xlsx file contains all computationally and experimentally obtained optical spectra data as well as the initial positions for all MD calculations.

## References

[1] Volkov S.V. (2012). Coordination Compounds in Melts. Molten Salts: From Fundamentals to Applications.

[2] Sridharan K., Allen T.R. (2013). Corrosion in Molten Salts. Molten Salts Chemistry.

[3] Li S.X. (2002). Anodic Process of Electrorefining Spent Nuclear Fuel in Molten LiCl-KCl-UCl3/Cd System. ECS Proc. Vol..

[4] Zhang J. (2014). Electrochemistry of actinides and fission products in molten salts—Data review. J. Nucl. Mater..

[5] Barbanel Y.A., Kolin V.V., Kotlin V.P., Lumpov A.A. (1990). Coordination chemistry of actinides in molten salts. J. Radioanal. Nucl. Chem. Artic..

[6] Harrison T.J., Holcomb D.E., Flanagan G.F., Patton B.W., Gehin J.C., Howard R.L. (2011). Fast Spectrum Molten Salt Reactor Options.

[7] Fujii T., Nagai T., Sato N., Shirai O., Yamana H. (2005). Electronic absorption spectra of lanthanides in a molten chloride: II. Absorption characteristics of neodymium(III) in various molten chlorides. J. Alloys Compd..

[8] Yatsimirskii K.B. (1977). Spectroscopic studies on coordination compounds formed in molten salts. Non-Aqueous Solutions–5.

[9] Phillips W.C., Gakhar R., Horne G.P., Layne B., Iwamatsu K., Ramos-Ballesteros A., Shaltry M.R., LaVerne J.A., Pimblott S.M., Wishart J.F. (2020). Design and performance of high- temperature furnace and cell holder for in situ spectroscopic, electrochemical, and radiolytic investigations of molten salts Design and performance of high-temperature furnace and cell holder for in situ spectroscopic. Rev. Sci. Instrum..

[10] Smith G.P., James D.W., Boston C.R. (1965). Optical Spectra of Tl^+^, Pb^2+^, and Bi^3+^ in the Molten Lithium Chloride—Potassium Chloride Eutectic. J. Chem. Phys..

[11] Brynestad J., Boston C.R., Smith G.P. (1967). Electronic spectra and coordination of nickel centers in liquid lithium chloride-potassium chloride mixtures. J. Chem. Phys..

[12] Smith W.E., Brynestad J., Smith G.P., Smith W.E., Brynestad O., Smitht G.P. (1970). Spectroscopic Behavior and Coordination of Nickel(II) in Liquid Mixtures of Zinc and Cesium Chlorides Spectroscopic Behavior and Coordination of Nickel(II) in Liquid Mixtures of Zinc and Cesium Chlorides. J. Chem. Phys..

[13] Gruen D.M., Division C. (1967). Octahedral and tetrahedral co-ordination states of cobalt (II) in molten zinc chloride—aluminium chloride mixtures. J. Inorg. Nucl. Chem..

[14] Gruen D.M., McBeth R.L. (1963). The coordination chemistry of 3d transition metal ions in fused salt solutions. Pure Appl. Chem..

[15] Brookes H.C., Flengas S.N. (1970). Spectroscopy of chromium compounds in molten salts. Can. J. Chem..

[16] Jørgensen C.K. (1958). Ligand field bands of four-coordinated paramagnetic nickel(ii) complexes. Mol. Phys..

[17] Cho Y.H., Kim T.J., Park Y.J., Im H.J., Song K. (2010). Electronic absorption spectra of Sm(II) and Yb(II) ions in a LiCl-KCl eutectic melt at 450 °C. J. Lumin..

[18] Banks C.V., Heusinkveld M.R., O’laughlin J.W. (1961). Absorption Spectra of the Lanthanides in Fused Lithium Chloride—Potassium Chloride Eutectic. Anal. Chem..

[19] Kim B.Y., Yun J.I. (2016). Optical absorption and fluorescence properties of trivalent lanthanide chlorides in high temperature molten LiCl-KCl eutectic. J. Lumin..

[20] Yang L., Cohen M.L., Louie S.G. (2007). Excitonic effects in the optical spectra of graphene nanoribbons. Nano Lett..

[21] Marini A., Del Sole R. (2003). Dynamical excitonic effects in metals and semiconductors. Phys. Rev. Lett..

[22] Kunz B., Flynn C.P. (1983). Excitonic effects in the interband spectra of metals. Phys. Rev. Lett..

[23] Wang H. (2017). Excitonic effects and optical spectra of graphene nanoflakes. J. Appl. Phys..

[24] Kresse G. (1995). Ab initio molecular dynamics for liquid metals. J. Non. Cryst. Solids.

[25] Kresse G., Hafner J. (1994). Ab initio molecular-dynamics simulation of the liquid-metalamorphous- semiconductor transition in germanium. Phys. Rev. B.

[26] Kresse G., Furthmüller J. (1996). Efficiency of ab-initio total energy calculations for metals and semiconductors using a plane-wave basis set. Comput. Mater. Sci..

[27] Kresse G., Furthmüller J. (1996). Efficient iterative schemes for ab initio total-energy calculations using a plane-wave basis set. Phys. Rev. B—Condens. Matter Mater. Phys..

[28] Perdew J.P., Burke K., Ernzerhof M. (1996). Generalized gradient approximation made simple. Phys. Rev. Lett..

[29] Ernzerhof M., Perdew J.P., Burke K. (1997). Coupling-constant dependence of atomization energies. Int. J. Quantum Chem..

[30] Momma K., Izumi F. (2011). VESTA 3 for three-dimensional visualization of crystal, volumetric and morphology data. J. Appl. Crystallogr..

[31] Gajdoš M., Hummer K., Kresse G., Furthmüller J., Bechstedt F. (2006). Linear optical properties in the projector-augmented wave methodology. Phys. Rev. B—Condens. Matter Mater. Phys..

[32] Wang V., Xu N., Liu J.-C., Tang G., Geng W.T. (2021). VASPKIT: A Pre-and Post-Processing Program for VASP code. Comp. Phys. Commun..

[33] Heyd J., Scuseria G.E., Ernzerhof M. (2003). Hybrid functionals based on a screened Coulomb potential. J. Chem. Phys..

[34] Gill S.K., Huang J., Mausz J., Gakhar R., Roy S., Vila F., Topsakal M., Phillips W.C., Layne B., Frenkel A.I. (2020). Connections between the Speciation and Solubility of Ni(II) and Co(II) in Molten ZnCl_2_. J. Phys. Chem. B.

[35] Greenberg J., Sundheim B.R. (1958). Absorption spectra in molten salt solutions. J. Chem. Phys..

[36] Sundheim R., Harrington G. (1959). Absorption spectrum of NiCl_2_ in molten LiCl/KCl. J. Chem. Phys..

[37] Gabriel J., Vincent D., Bouteillon J., Poignet J., Volkovich V., Griffiths T. (1999). Molybdenum chemistry in molten LiCl–KCl eutectic: An electrochemical and absorption spectroscopy study of the concentration dependent stability of solutions of K_3_MoCl_6_. Electrochim. Acta.

[38] Brevnova N.P., Polovov I.B., Chernyshov M.V., Volkovich V.A., Vasin B.D., Griffiths T.R. (2013). Electronic Absorption Spectra of Niobium Species in Halide Melts. ECS Trans..

