# Supplementary material for: Local Coordination Environment of 3d and 4d Transition Metal Ions in LiCl-KCl Eutectic Mixture
Source: Materials (Basel). 2022 Feb 16;15(4):1478. doi: 10.3390/ma15041478 (PMC8877717; doi:10.3390/ma15041478)
Supplement: Supplementary file 1 [file materials-15-01478-s001.zip › materials-1543691-supplementary.pdf]

materials

MDPI

Supplemental Information

Local Coordination Environment of 3d and 4d Transition Metal ions in LiCl-KCl Eutectic Mixture

Jon Fuller[1,2], William Phillips[1], Qi An[2*], and Ruchi Gakhar[1*]

Idaho National Laboratory, Pyrochemistry and Molten Salt Systems Department, Idaho, NV 83415, USA; jon-fuller@nevada.unr.edu (J.F.); william.phillips@inl.gov (W.P.)

[2] Department of Chemical and Materials Engineering, University of Nevada, Reno, NV 89577, USA

* Correspondence: qia@unr.edu (Q.A.); ruchi.gakhar@inl.gov (R.G.)

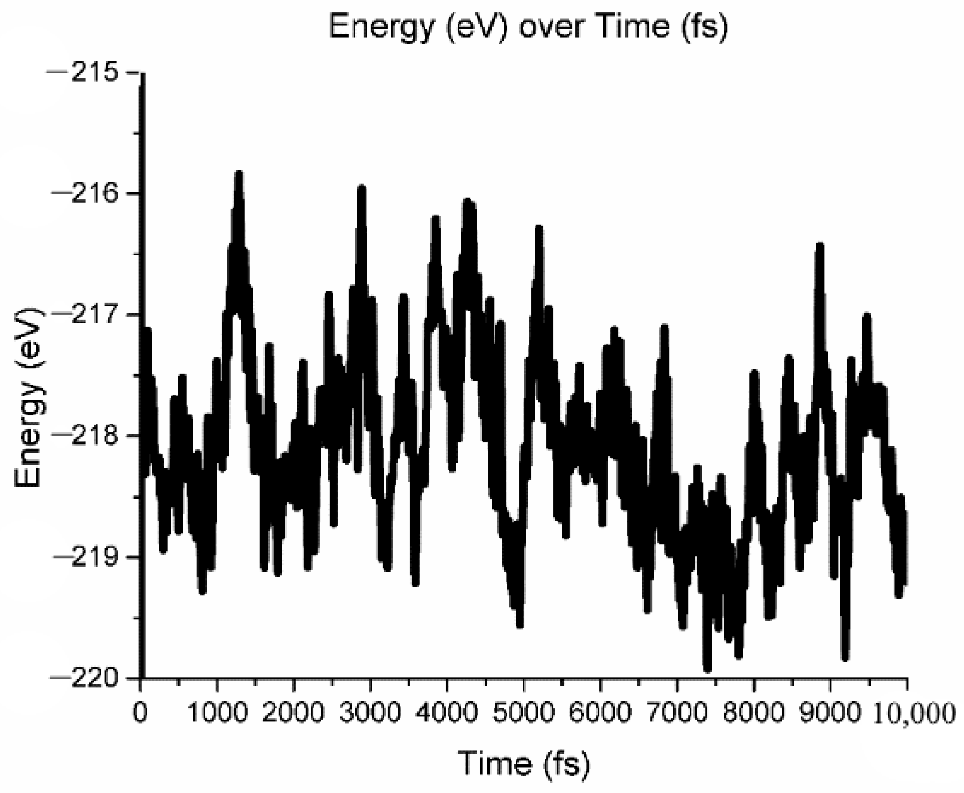

Figure S1. Energy convergence of Ni^{2+} in the LiCl-KCl eutectic composition at the target temperature of 400 °C after an extended period of equilibration (only the final 10 ps are shown). Energy of system converged at average value of −218.2 eV.

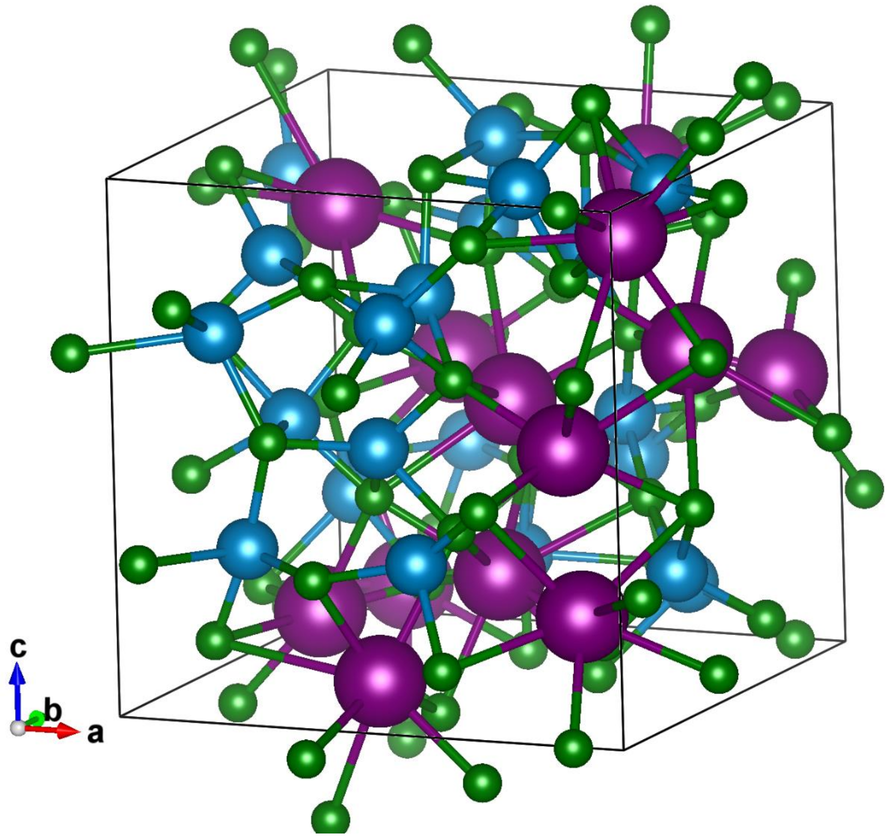

Figure S2. The computationally obtained LiCl-KCl eutectic composition from molecular dynamics simulations at 400 °C. Blue spheres, green spheres and purple spheres represent Li, Cl and K atoms, respectively.

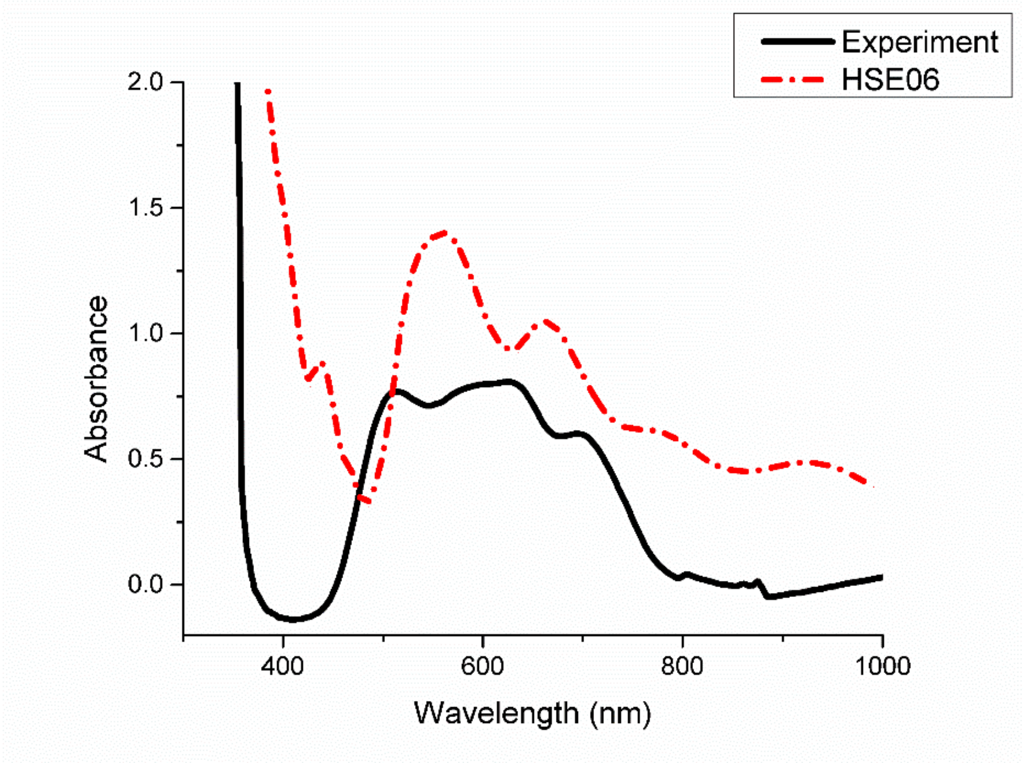

Figure S3. The computationally obtained Ni^{2+} spectrum obtained using the HSE-06 hybrid functional overlaid on the experimentally obtained Ni^{2+} optical spectrum at 400 °C.
